# Leveraging Synteny to Generate Reference Genomes for Conservation: Assembling the Genomes of Hector's and Māui Dolphins

**DOI:** 10.1111/1755-0998.14109

**Published:** 2025-04-04

**Authors:** S. Alvarez‐Costes, C. S. Baker, R. Constantine, E. L. Carroll, J. Guhlin, L. Dutoit, S. Ferreira, D. Heimeier, N. J. Gemmell, J. Gillum, R. M. Hamner, W. Rayment, W. Roe, B. Te Aikā, L. Urban, A. Alexander

**Affiliations:** ^1^ Department of Anatomy, School of Biomedical Sciences University of Otago Dunedin New Zealand; ^2^ Marine Mammal Institute and Department of Fisheries, Wildlife, and Conservation Sciences Oregon State University Newport Oregon USA; ^3^ School of Biological Sciences University of Auckland–Waipapa Taumata Rau Auckland New Zealand; ^4^ Department of Biochemistry School of Biomedical Sciences, University of Otago Dunedin New Zealand; ^5^ Department of Zoology University of Otago Dunedin New Zealand; ^6^ Department of Marine Science University of Otago Dunedin New Zealand; ^7^ School of Veterinary Science Massey University Palmerston North New Zealand; ^8^ Research and Enterprise University of Otago Dunedin New Zealand; ^9^ Helmholtz AI and Helmholtz Pioneer Campus, Helmholtz Munich Neuherberg Germany; ^10^ School of Life Sciences Technical University of Munich Freising Germany

**Keywords:** *Cephalorhynchus hectori*, conservation genomics, heterozygosity, historical demography, reference genomes, synteny

## Abstract

Escalating concern regarding the impacts of reduced genetic diversity on the conservation of endangered species has spurred efforts to obtain chromosome‐level genomes through consortia such as the Vertebrate Genomes Project. However, assembling reference genomes for many threatened species remains challenging due to difficulties obtaining optimal input samples (e.g., fresh tissue, cell lines) that can characterise long‐term conservation collections. Here, we present a pipeline that leverages genome synteny to construct high‐quality genomes for species of conservation concern despite less‐than‐optimal samples and/or sequencing data, demonstrating its use on Hector's and Māui dolphins. These endemic New Zealand dolphins are threatened by human activities due to their coastal habitat and small population sizes. Hector's dolphins are classified as endangered by the IUCN, while the Māui dolphin is among the most critically endangered marine mammals. To assemble reference genomes for these dolphins, we created a pipeline combining *de novo* assembly tools with reference‐guided techniques, utilising chromosome‐level genomes of closely related species. The pipeline assembled highly contiguous chromosome‐level genomes (scaffold N50: 110 MB, scaffold L50: 9, miniBUSCO completeness scores > 96.35%), despite non‐optimal input tissue samples. We demonstrate that these genomes can provide insights relevant for conservation, including historical demography revealing long‐term small population sizes, with subspecies divergence occurring ~20 kya, potentially linked to the Last Glacial Maximum. Māui dolphin heterozygosity was 40% lower than Hector's and comparable to other cetacean species noted for reduced genetic diversity. Through these exemplar genomes, we demonstrate that our pipeline can provide high‐quality genomic resources to facilitate ongoing conservation genomics research.

## WHAKARāPOPOTO

Kua toko ake te āwangawanga mō ngā momo mōrea nā te mimiti haere o te kanorau o ō rātou huinga ira. Me te aha, e kitea ana te katoa o ngā pūira i tēnā i tēnā huinga ira e ngā rōpu pērā i te “Vertebrate Genomes Project”. Heoi anō, he uaua te kitenga o te huinga ira tohutoru i te marea o ngā momo mōrea nā te wero hei kohikohi i ngā tīpako pai (e.g., ko te tīpako pūtautau hou, ko te pūtau whakatipu). He māori taua āhuatanga i ngā kohikohinga e aro ai ki te whāomoomo. Nā mātou tētahi anga i hanga hei whakamahi i te ōritetanga i ngā huinga ira o ngā momo whanaunga. Ahakoa te paku hēhē o ngā tīpako, o ngā raraunga pītau ira nōki e whakamahi ai, mā taua anga te huinga ira tino pai kē e puta. Kua whakamahia taua anga kia kitea ai te huinga ira o ētahi ika moana, arā, ko te popoto me te tūpoupou (e maha ngā ingoa o tēnei kararehe, ko ‘tūpoupou’ te ingoa e whakamahia ai i tēnei tuhinga). Nā tātou, arā, nā ngā tāngata ēnei ika moana e mōrearea ai nā te mea e noho ana rātou i te takutai, ā, torutoru noa iho ngā kararehe ki tēnā, ki tēnā o ngā taupori. E ai ki te IUCN, e “endangered” ana te tūpoupou, heoi, ko te mūtunga kē mai nei o te ika moana e “critically endangered” ai ko te popoto. E kitea ai te hunga ira tohutoru o ēnei ika moana ka rānga ngā taputapu *de novo* me ngā mea tohutoru e whakamahi ai i ngā huinga ira nō ngā momo whanaunga kua kitea te katoa o ngā pūira i te huinga ira. Ahakoa te paku hēhē o ngā tīpako pūtautau, nā tā mātou anga ngā pūira katoa o te huinga ira i kite, ā, he tukupū hoki (tīrewa N50: 110 MB, tīrewa L50: 9, tā miniBUSCO tatau tutuki > 96.35%). Kua whākina ngā hua e hāngai ana ki te whāomoomo e ēnei huinga ira tae ana ki te ititinga o ngā taupori mai rā anō, ā, i ~ 20kya te whakawehewehe o te taupori tūpoupou me te taupori popoto, ākene pea nā te mahi a te wā o te tio nui mutunga. Ko te kanorau o tō te popoto huinga ira, he 40% o te kanorau o tō te tūpoupou. He ōrite te kanorau o tō te popoto ki ētahi atu ika moana e iti rawa ai te kanorau. E whāki ana ēnei huinga ira hou ka taea e tēnei anga te kite ngā rauemi huinga ira kia tautoko ai i te rangahau whāomoomo. Whaowhina te kete mātauranga!

## 1 Introduction

Assembly of reference genomes for threatened species presents a challenge primarily due to the limited availability of high‐quality tissue samples, resulting in a lack of high molecular weight DNA for sequencing (Formenti et al. 2022). Therefore, these assemblies tend to be represented by many non‐assigned scaffolds, making these genomes highly fragmented and not at chromosomal level (Totikov et al. 2021). Whales, dolphins and porpoises (cetaceans) being fully aquatic mammals, are particularly hard to sample from free‐swimming, living individuals, as they spend most of the time underwater and can be elusive. This leaves many species to be represented in tissue archives solely by stranded or beachcast individuals, subjected to variable levels of decomposition, in addition to the decomposition that results from non‐suitable storage when sampling in remote places. Exceptions to this include high‐quality cetacean chromosome‐level genomes obtained from species kept in captivity, which facilitates access to high‐quality tissue and the development of cell lines (e.g., bottlenose dolphin, *Tursiops truncatus*, and killer whale, *Orcinus orca* ) (Foote et al. 2015; Foote and Bunskoek 2022). However, animal welfare concerns preclude this pathway for many species. For example, the vaquita ( *Phocoena sinus* ) genome (Vertebrate Genomes Project [VGP]) was generated from an individual that died during capture in an attempt to establish a captive insurance population for this critically endangered species (Morin et al. 2021). Researchers were able to sample tissue from different organs and perform cell cultures to obtain high‐quality DNA (10× and Hi‐C data), making the most of this unfortunate event, which also marked the end of efforts to establish a captive population.

Given the importance of “platinum‐standard” reference‐quality genomes to enable conservation genomics (Morin et al. 2020), there is a need to generate high‐quality genomes from existing long‐term tissue archives of species of conservation concern, which may not have been stored in a manner optimal for current best‐practice assembly techniques (e.g., at temperatures ≤ − 80**°**C). Here, we present a pipeline that leverages genome synteny within a taxonomic group to assemble high‐quality genomes despite less‐than‐optimal tissue and sequence inputs. Cetaceans exhibit remarkable karyotype conservation, ranking among the most conserved of all mammal orders (Árnason et al. 1984; Ferguson‐Smith and Trifonov 2007). The high level of chromosomal stability supports the use of synteny‐based approaches for genome assembly within this group. We demonstrate the efficacy of this approach using the endangered Hector's ( *Cephalorhynchus hectori hectori* ) and the critically endangered Māui (*C. h. maui*) dolphins, endemic to Aotearoa New Zealand waters.

Until recently, Hector's and Māui dolphins were considered a single species but were re‐classified as subspecies in 2002 based on cranial morphology and genetic differences (Baker et al. 2002; Slooten 1990). Small size and inshore distribution make these dolphins very vulnerable to anthropogenic threats (e.g., fisheries bycatch, pollution, marine traffic, disease) (Roberts et al. 2020; Roe et al. 2013, 2017; Slooten et al. 2006; Slooten and Dawson 2021). However, the two subspecies differ significantly in their threatened status. There are ~15,000 Hector's dolphins distributed around *Te Waipounamu* (the South Island of Aotearoa New Zealand), currently classified as endangered by the IUCN (Braulik et al. 2023; Mackenzie and Clement 2016; Slooten and Dawson 2021). In contrast, with only 48 individuals older than 1 year old (2020–2021 census) and reduced genetic diversity, the Māui dolphin is one of the most endangered cetaceans in the world, being listed as critically endangered by the IUCN (Constantine et al. 2021; Dawson and Slooten 2001; Pichler and Baker 2000).

Previous research has identified an erosion of genetic diversity in the Māui dolphin due to declines associated with historical fishing pressure (Pichler and Baker 2000). This is concerning given genetic diversity is essential to adapt to changing environmental conditions and selective pressures (Ellegren and Galtier 2016; Hoffmann and Sgró 2011). Despite the distinct geographic distributions of Hector's and Māui dolphins, which are believed to be influenced by *Te Moana o Raukawa* (The Cook Strait) serving as a deep‐water barrier due to the species' preference for shallow waters (Bräger et al. 2003; Hamner et al. 2014; Rayment et al. 2010, 2011), there has been genetic confirmation of Hector's dolphins within the Māui dolphin range. This suggests the potential for gene flow via these migrants into the Māui dolphin population. Yet, no ‘hybrids’ between Hector's dolphin migrants and Māui dolphins have been identified, meaning the implications for the genetic diversity and fitness of Māui dolphins remain uncertain, which could include the risks associated with outbreeding depression (Hamner et al. 2014).

Increasing concern about the impacts of reduced genetic diversity on endangered species such as Hector's and Māui dolphins has spurred efforts to obtain chromosome‐level genomes of vertebrates through consortiums such as the G10K and Vertebrate Genomes Project (VGP) (Rhie et al. 2021). High‐quality genomes generated using combinations of technology such as short reads, long reads and Hi‐C technology (Chakraborty et al. 2016; Totikov et al. 2021) allow the assessment and visualisation of the distribution of heterozygosity along the different chromosomes of a genome. This information can also be compared between different species and populations to detect genomic regions affected by evolutionary forces such as natural selection, genetic drift, inbreeding and introgression (Stange et al. 2021; Totikov et al. 2021), thereby guiding conservation strategies that protect genome‐wide diversity and reduce the likelihood of species extinction (Hoelzel et al. 2019; Mable 2019).

Here we present a novel pipeline for leveraging genome synteny within taxonomic groups by combining *de novo* assembly tools with reference‐guided tools using available chromosome‐level genomes from closely related species, applied to Hector's and Māui dolphins (https://github.com/sebasalco/GenomeSynteny_HectorsMauiRefGenomes). The resultant high‐quality genomes enable conservation actions by providing a reference to assess kinship and inbreeding, and by beginning to unravel the demographic history and evolutionary dynamics of both Hector's and Māui dolphins. We demonstrate that by utilising high‐quality genomes of closely related species, despite limited‐quality samples, we can obtain high‐quality genomes that can enable conservation genomics.

## 2 Methods

### 2.1 Sample Collection and DNA Extraction

A representative individual was selected for each of the two subspecies based on available tissue amounts: a male Hector's dolphin (University of Auckland biopsy sample ID: Che11CB067, also biopsied as Che11CB069, Che12CB071, and Che12CB089) biopsied four times between 2011 and 2012 in *Te Koko‐o‐Kupe* (Cloudy Bay), *Te Waipounamu* (South Island) (Hamner et al. 2017), and a female Māui dolphin (NZCeTA sample ID: Chem18NZ02 (U18‐042); DOC animal ID: H273), stranded north of *Whaingaroa* (Raglan), *Te‐Ika‐a‐Māui* (North Island) of New Zealand. Consultation with *iwi* (Māori tribes) who had *rangatiratanga* and *kaitiakitanga* (sovereignty and responsibilities) for the area where the samples were obtained occurred regarding the overall aims of the study (providing conservation genomic resources for Hector's and Māui dolphins) and the location and *tikanga* (protocols) of the research. These dolphins have important roles as *kaiārahi* (guides) for the great voyaging *waka* (canoes) that travelled to Aotearoa and for *wairua* (spirits) of those who have passed returning to *Hawaiki* (the ancestral homeland). As such, they and their data are both *taonga* (treasured) and *tapu* (restricted), and treating them with respect is paramount. There was a preference for tissues to remain in Aotearoa New Zealand to be consistent with rights and responsibilities (particularly *kaitiakitanga*) under *Te Tiriti o Waitangi* (the founding document of New Zealand). Therefore, all DNA extraction and library preparation was conducted within New Zealand. DNA of the representative individuals for Chromium 10× linked‐reads sequencing and Nanopore sequencing was extracted from ~30 mg of skin/blubber interface tissue using the Circulomics Nanobind Tissue Big DNA kit (Guide & overview—Nanobind tissue kit v1.0 11/19) with the manufacturer's recommendation for ethanol removal and tissue homogenization utilising a Qiagen TissueRuptor II (with modification to utilise 2 mL tubes). BluePippin was used to further select high molecular weight DNA (> 40 kbp). Because samples had been stored between −20**°**C and 4**°**C in ethanol, resultant sample quality meant multiple DNA extractions and size selection with BluePippin were necessary to reach input quality/quantity for library preparation. Additionally, DNA extractions for Illumina whole genome sequencing (WGS) of the reference individuals were performed using the ZymoBIOMICS DNA Miniprep Kit, following the manufacturer's protocol, with the following modifications: before proceeding to bead‐bashing, samples were rinsed twice with 500 μL PCR‐grade H_2_O to remove excess ethanol, and then macerated by scalpel. Samples were then homogenised with the Qiagen TissueRuptor II in a 2 mL tube with 120 μL H_2_O, 120 μL Solid Tissue Buffer (Blue) and 10 μL Proteinase K, before being incubated at 55**°**C overnight on a Thermomixer (99 rpm). Samples were bead‐bashed for 40 min before proceeding with the protocol.

### 2.2 Library Preparation and Sequencing

Libraries were prepared from high molecular weight DNA using the 10× Genomics Chromium controller and genome linked‐read technology for each individual, following the manufacturer's protocol, followed by sequencing on a portion of an Illumina Novaseq S2 lane (alongside unrelated samples). Oxford Nanopore long‐read libraries were prepared using SQK‐LSK10 kits, and each sequenced on a separate FLO‐MIN106 MinION flow cell. We obtained DNA with higher molecular weight for the Hector's dolphin, as the sample was of better quality than for the Māui dolphin. We therefore sequenced six Nanopore libraries for the Māui dolphin, in contrast with two for the Hector's dolphin, in an attempt to compensate for the poorer DNA quality of the Māui dolphin. The DNA for the short‐read WGS for each individual was made into a PCR‐free library using the Tecan Rapid EZ DNAseq kit, following the manufacturer's protocol (M01515 v1.1). The Hector's and Māui dolphins short‐read WGS libraries were multiplexed with 52 other unrelated samples based on Kapa qPCR quantification and sequenced on an Illumina Novaseq S2 lane.

### 2.3 Reference Genome Assembly

**FIGURE 1:**
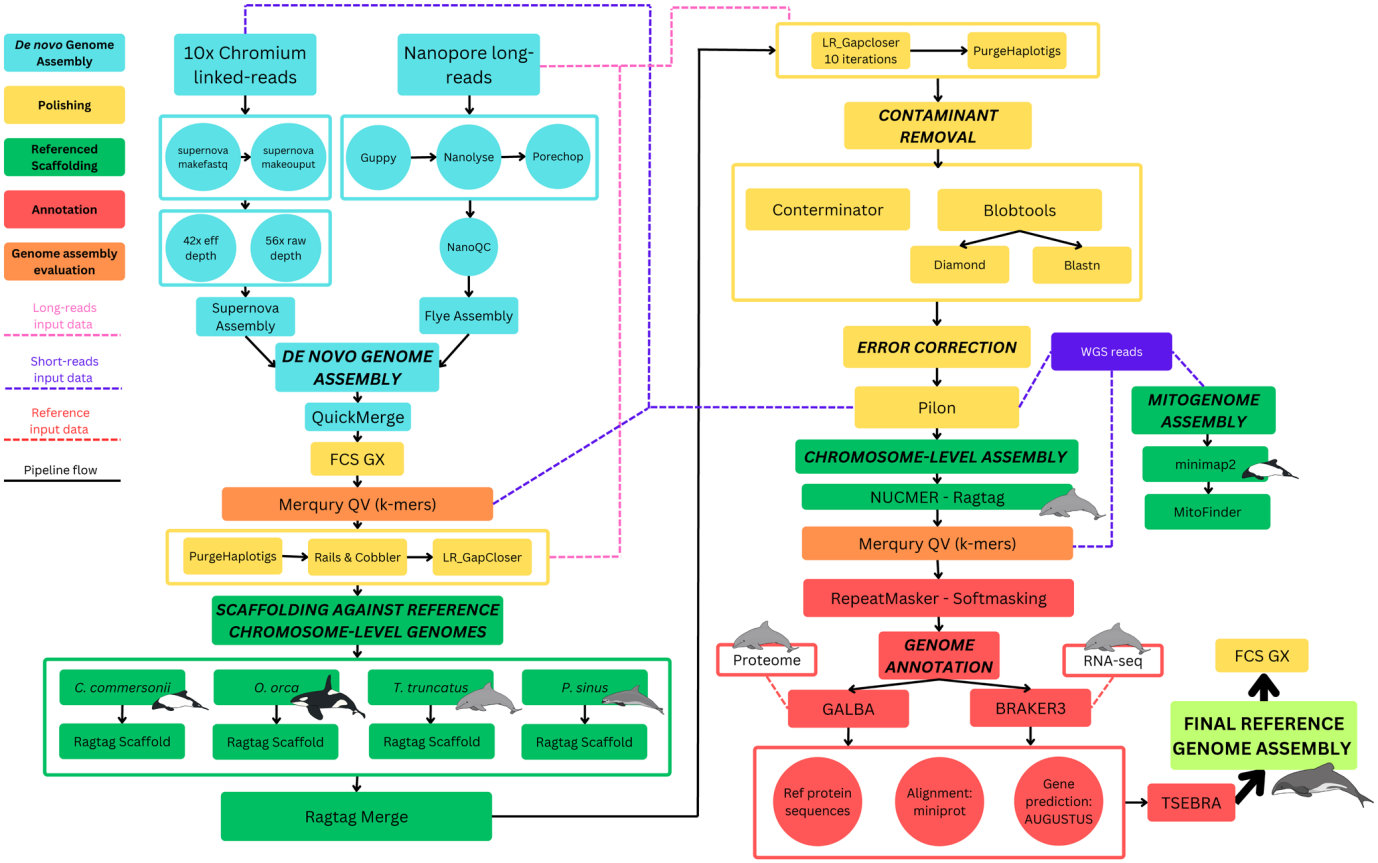
Schematic representation of the assembly pipeline used for the Hector's and Māui dolphin genomes. Blue represents *de novo* genome assembly, green represents referenced‐based scaffolding, yellow represents polishing steps and red represents genome annotation steps. Black arrows represent the pipeline flow and dotted arrows represent the input data used in each of the polishing steps. The general steps are given in uppercase bold letters, with the programs in lowercase letters. The specific code to achieve these steps is located at https://github.com/sebasalco/GenomeSynteny_HectorsMauiRefGenomes.

Reference genome assembly was carried out using two general approaches: a *de novo* genome assembly approach using data from two different technologies (10× Genomics Chromium Linked‐Reads and Nanopore Long‐Reads) and a reference scaffolding approach described in greater detail below (Figure 1).

The 10× Genomics Chromium Linked‐Reads were assembled using Supernova (v2.1.1) (Weisenfeld et al. 2017). First, we wrapped and demultiplexed the sequences using Supernova mkfastq. The assembly for each dolphin was performed using Supernova with default parameters and without specific—maxreads in order to utilise all the available reads for both of our assemblies. Following the Supernova run, we extracted the two pseudohaploid genomes for each run using Supernova mkoutput. Genome size was estimated based on the available reference genomes of other dolphin species and also on estimates from the Supernova assembler.

For the Nanopore assemblies, base calling, barcode/adapter trimming, and quality control analyses were performed separately on each of the two Hector's dolphin runs and each of the six Māui dolphin runs. Guppy (v6.4.6) was used to base call using GPU base‐calling (Wick et al. 2019). Adapters and barcodes were removed using Porechop (v0.2.4) and Nanolyse (v1.2.0) was used to identify and remove control lambda DNA in comparison to the default reference lambda genome (De Coster et al. 2018; Wick et al. 2017). Following quality control assessment with pycoQc (v2.5.2) (Leger and Leonardi 2019), the genome for each dolphin was assembled with Nanopore reads using Flye (v2.9.1) assuming an estimated genome size of 2.3 Gbp with three polishing iterations (Kolmogorov et al. 2019).

To obtain high‐quality genomes with higher contiguity, the 10× and Nanopore assemblies were merged for each dolphin using QuickMerge v0.3 (Chakraborty et al. 2016). For the Hector's dolphin, the Supernova assembly (Chromium 10× linked reads) had higher coverage, contiguity, and complete BUSCO scores compared to the Hector's dolphin's Flye assembly (Nanopore) and was therefore used as a reference genome for the merge. In contrast, for the Māui dolphin the Flye assembly (Nanopore) had higher coverage, contiguity, and complete BUSCO scores than the Supernova assembly, so it was used as a reference for the Supernova assembly. All BUSCO scores were estimated using miniBUSCO (Huang and Li 2023a, 2023b). We estimated BUSCO scores after assembly merging and at the end of the complete assembly process. We also assessed the completeness and error rate of the assemblies using the k‐mer‐based Merqury QV approach (v1.3) (Rhie et al. 2020). First, we generated a meryl database from the short‐read WGS for each assembly. The analysis was conducted both before the polishing process and after obtaining the final assembly to evaluate if there was any artefact of the polishing process on accuracy and completeness.

Merged assemblies went through several stages of additional polishing using raw Nanopore reads. For the first polishing stage, unpaired allelic contigs were eliminated using Purgehaplotigs (v1.1.2), which also helps filter out spurious mitochondrial‐derived sequences that may have been incorporated into the nuclear assembly (v1.1.2) (Roach et al. 2018) and gaps were closed using Rails (v1.1.5), Cobbler (v0.6.1) (Warren 2016), and LR_GapCloser with 10 iterations (Xu et al. 2018). The second polishing stage was performed using the Supernova assemblies to gap close and scaffold the genomes with RagTag (v2.1.0) (Alonge et al. 2022).

We used a chromosome‐length genome (defined as a genome assembly that includes the entire length of the genome, but not necessarily with the entirety of all chromosomes represented by DNA sequence) of the Commerson's dolphin ( *Cephalorhynchus commersonii* , https://www.dnazoo.org/assemblies/cephalorhynchus_commersonii), and four chromosome‐level reference genomes (defined as a complete genome in which the genome sequence is organised and assigned to individual chromosomes) of other cetacean species to scaffold the assemblies, vaquita (*Phocoena sinus*, NCBI GCF_008692025.1), orca ( *Orcinus orca* , NCBI GCF_937001465.1), bottlenose dolphin ( *Tursiops truncatus* , NCBI GCF_011762595.1), and blue whale ( *Balaenoptera musculus* , NCBI GCF_009873245.2). We utilised the Commerson's dolphin chromosome‐length genome for the first round of scaffolding because this species is in the same genus as the Hector's and Māui dolphin. Further scaffolding was performed against the remaining higher quality chromosome‐level genomes using the RagTag “scaffold” module, resulting in a total of five different scaffoldings for each dolphin. A consensus scaffold was obtained with the “merge” option of RagTag using the scaffold outputs of the previous step. Draft scaffolded genomes were passed through another round of polishing with three iterations of gap filling with LR_GapCloser and polishing and error correction using short‐read data with Pilon (v1.24) (Walker et al. 2014).

To obtain the final assemblies, contaminants were removed using Conterminator (Steinegger and Salzberg 2020) and Blobtools (v4.1.4), comparing the assemblies against the NCBI nucleotide and Diamond Uniprot databases (Laetsch and Blaxter 2017). Chromosome‐level assemblies were reached after a final alignment and orientation of the draft genomes against the bottlenose dolphin genome, utilising RagTag and NUCMER (MUMmer 4.0.0) (Marçais et al. 2018). A final miniBUSCO assessment was then performed against the Cetartiodactyla database to evaluate the quality of the assemblies (Huang and Li 2023a, 2023b). We used FCS‐GX (Astashyn et al. 2024) to screen our final genome assemblies for contamination and taxonomic composition to ensure that all remaining contamination, including adaptors and mitochondrial sequences, was removed (Figure S1).

To evaluate the impact of reference scaffolding on the mapping quality of long reads, we assessed the alignment of Nanopore reads both before and after scaffolding. We aligned the raw ONT reads to the pre‐scaffolded genome assemblies and the final genome assemblies using minimap2 (v2.24) (Li 2018). Initially, we created minimap2 index files for both the pre‐scaffolded and final assemblies. Following this, we aligned the ONT reads to these indexed genomes using minimap2 ‐ax map‐ont, then sorted and indexed the resulting BAM files with SAMtools. We assessed mapping quality using samtools flagstat to compare alignment statistics between the pre‐scaffolded and scaffolded genome assemblies (Supp. Table 1).

**TABLE 1:** Genome annotation summary for the Hector's and Māui dolphin genomes, in comparison with selected chromosome‐level cetacean genome annotations.

Species	Genes	Total mRNAs	Total introns	Total exons	Total CDS
Hector's	22,001	42,256	171,569	149,568	42,315
Māui	20,721	39,569	132,469	111,750	39,600
Bottlenose	25,638	55,726	227,256	259,958	55,739
Killer whale	27,865	63,196	239,323	271,718	63,209
Vaquita	23,307	52,282	217,934	249,232	52,320
Blue Whale	24,213	52,246	219,171	246,134	52,259

We created synteny plots of the Hector's and Māui dolphin final assemblies against the bottlenose dolphin reference genome, as well as a synteny plot of the blue whale genome against the vaquita genome and the vaquita genome against the bottlenose dolphin genome to demonstrate broader patterns of synteny within Cetacea. The Commerson's dolphin genome was not included in this analysis due to a lack of contiguity, being found in > 1000 scaffolds. To construct the synteny plots, we conducted whole‐genome alignments using minimap2 (v2.24) (Li 2018). Using coordinates output by minimap2, we carried out the synteny analysis with NGenomeSyn (v1.41) (He et al. 2023).

The mitochondrial genomes were extracted with MitoFinder (v1.4.1) (Allio et al. 2020), using the WGS and 10× short reads mapped against the mitogenome of the closely related Commerson's dolphin (NCBI NC_060610.1). We assessed the coverage of the mitogenomes by mapping the short reads against the obtained mitogenomes with minimap2 (v2.24) (Li 2018), extracting a bam file using samtools (v.1.16.1) (sort ‐@10 ‐O BAM) and getting the read depth with bedtools (v2.30) (genomecov). The mitogenomes were aligned with one another and with the Commerson's dolphin mitogenome. One spurious 141 bp duplication in Hector's dolphin (in ND1) and another spurious 141 bp duplication in the Māui dolphin mitogenome (in the 16S rRNA) were removed after analysing coverage and finding that it was extremely low in these regions (likely the result of assembly error). The individual haplotype identity of the two individuals was confirmed by aligning the mitogenomes against the D‐loop database from Hamner et al. (2014).

### 2.4 Phylogenetic Analysis

To assess the phylogenetic relationships and accuracy of the assembled Hector's and Māui dolphin genomes, we constructed a phylogenetic tree using the SANS serif ambages (Abundance‐filter, Multi‐threading and Bootstrapping on Amino‐acid or GEnomic Sequences) module with 100 bootstraps (Rempel and Wittler 2021). SANS constructs phylogenetic trees using full genomes with a pangenomic k‐mer‐based approach, without alignment. The phylogenetic tree was constructed using the Hector's and Māui dolphin genomes alongside the chromosome‐level reference genomes used for the reference‐based scaffolding approach and synteny plots. A second tree was also constructed using the chromosome‐level and scaffold‐level genomes of all the Delphinidae species available in NCBI, with two porpoise species as an outgroup (Figure S2).

### 2.5 Genome Annotation

Before genome annotation, both genomes were screened for repetitive elements and softmasked using RepeatMasker (v4.1.0) against the Dfam database (Smit et al. 2023). In the absence of RNAseq availability for Hector's and Māui dolphins, we annotated the genomes using GALBA (v1.0.7) (Brůna et al. 2023) and BRAKER3 (v3.0.3) (Hoff et al. 2019), with a reference‐guided homology approach using the proteome and RNA‐Seq of the bottlenose dolphin, *Tursiops truncatus* (NCBI GCF_011762595.1 and NCBI PRJDB16986). This species was selected because it has the best proteome available for any dolphin species. GALBA annotation was performed with the “miniprot” option for training and AUGUSTUS ab initio for gene prediction. Both annotations were merged using TSEBRA, filtering all single‐exon genes with no support by a start or stop codon hint in the reading frame (Gabriel et al. 2021). The quality assessment of the annotation was performed using the miniBUSCO “proteome” option and GeneValidator (v2.1.12) (Dragan et al. 2016). Functional annotation and gene ID assignment were performed using eggNOG emapper.py (v2.1.12) (Cantalapiedra et al. 2021).

### 2.6 Historical Demography Reconstruction

The historical demography of the Hector's and Māui dolphin was reconstructed using the Pairwise Sequentially Markovian Coalescent (PSMC) model (v0.6.5) (Li and Durbin 2011) by obtaining an autosomal diploid fasta file for each dolphin, generated by obtaining variants by mapping WGS short reads to autosomal chromosomes of the reference genomes. Each dolphin's reads were mapped against their own reference genome (Hector's‐Hector's, Māui‐Māui) to avoid any reference bias (Cahill et al. 2016; Li and Durbin 2011). We mapped the reads using BWA (v0.7.15) (Li and Durbin 2009) and extracted a file for each alignment, obtaining a pileup file with a base quality filter using bcftools mpileup (v1.16) (‐Q 30 ‐q 30). We then called the variants with bcftools (v1.16) (‐c). We filtered sites with < 5× coverage, a maximum coverage of 34× and a base quality of 30 with vcfutils.pl vcf2fq (‐d 5 ‐D 35 ‐Q 30) transformed them into PSMC files (Li 2011; Li and Durbin 2009). Although PSMC analysis with low‐coverage whole genome sequencing data has previously proved successful (Cooper et al. 2020), by excluding sites with coverage below 5× there is an improvement in model accuracy (Cheng et al. 2014). This filter can help combat previous observations that low coverage can affect and flatten the magnitude of PSMC demographic curves (Carroll et al. 2021), instead resulting in inferences comparable to those derived from high‐coverage datasets (Auton et al. 2015; Mather et al. 2020).

The PSMC plot was scaled using a generation time of 12.5 years, estimated for Hector's dolphin (Hamner et al. 2017; Taylor et al. 2007), and an autosomal mutation rate of 1.08 × 10^−8^ used for other cetaceans in previous studies (Morin et al. 2021; Westbury et al. 2023), derived from a previously determined mutation rate for odontocetes (Dornburg et al. 2012). The PSMC inference was carried out with the recommended input parameters suggested by Morin et al. (2021) including at least 10 recombinations inferred to occur after 20 rounds of iterations, (*p* = 8 + 23 × 2 + 9 + 1). We obtained 100 bootstrap samples of the PSMC to assess variance, splitting the analysis by chromosome for efficiency. Population structure can considerably influence the results of a PSMC analysis, particularly when comparing closely related individuals (e.g., from two subspecies) (Chikhi et al. 2010; Mazet et al. 2015, 2016). Therefore, to analyse when Hector's and Māui dolphins could have been reproductively isolated from each other (cessation of gene flow), we performed a pseudodiploid/hybrid PSMC by making a synthetic mixed genome between the Hector's and Māui dolphin, using seqtk to randomly sample a single allele from each subspecies at each site (Morin et al. 2018; Mather et al. 2020).

### 2.7 Genome‐Wide Heterozygosity

Genome‐wide heterozygosity was calculated as a proxy for genetic diversity using the mapped WGS reads utilised in the PSMC analysis (Hector's reads mapped to the Hector's dolphin genome, Māui reads mapped to the Māui dolphin genome) within Analysis of Next Generation Sequencing Data (ANGSD) (v0.935). Due to the low coverage of the WGS reads, we used ANGSD's genotype likelihoods to account for uncertainty in genotype calls (Korneliussen et al. 2014). Autosomal heterozygosity was computed with realSFS using allele frequencies (‐dosaf 1), a minimum quality of 20, a minimum map quality of 30, a SNP_pval of 1e‐6, and the GATK genotype likelihood model (‐GL 2). We then calculated genome heterozygosity (π) across each autosomal chromosome for each dolphin in non‐overlapping 50 kbp windows and 5 kbp steps using the ANGSD thetaStat option.

## 3 Results

### 3.1 Chromosome Level Genomes Merging de Novo and Reference‐Based Approaches

The Supernova assembly of the Hector's dolphin was more contiguous and of higher quality than the Supernova assembly of the Māui dolphin (Table S1). In contrast, the Nanopore Flye assembly of the Māui dolphin was more contiguous than the Nanopore Flye assembly of the Hector's dolphin (Table S1). Merging the Supernova assembly of 10× chromium linked short reads and the Nanopore long‐reads Flye assembly resulted in a high‐quality contig genome for both the Hector's and Māui dolphin, with both assemblies achieving ~88% BUSCO, and contig N50 above 1 Mbp (Table S1). The merged genome assemblies were carefully screened and cleaned for contamination using FCS‐GX. The merged assembly of Hector's dolphin exhibited a higher level of contamination (7 Mb) compared to the merged assembly of the Māui dolphin (2 Mb). In both cases, the predominant source of contamination was identified as β‐proteobacteria (Figure S1). Using the pipeline we developed here, further reference scaffolding against closely related chromosome‐level genomes resulted in a high‐quality contiguous chromosome‐level genome for the Hector's and Māui dolphin, with each of the polishing and scaffolding steps resulting in an improvement in the genome quality (Table S1). The final assembled genome size for the Hector's and Māui dolphin was 2.3 Gbp, with a scaffold N50 of 110 Mbp and scaffold L50 of 9 for both genomes. The mitogenomes obtained were 16,388 bp and provide a complete reference mitogenome for both subspecies (previously only protein‐coding genes and the D‐loop were available). The Hector's dolphin reference individual was confirmed as mitochondrial haplotype A and the Māui dolphin reference individual as haplotype G, the unique haplotype of the Māui dolphin population (Hamner et al. 2014).

The 2.3 Gbp genome was assembled into 339 scaffolds for the Hector's dolphin (male), with 99% (2,291,306,640 bp) assigned to the autosomal and X/Y sex chromosomes and < 1% in unassigned scaffolds (4,600,419 bp). For the Māui dolphin (female), the genome was assembled into 170 scaffolds, with 99% (2,308,694,106 bp) assigned to the 21 autosomal chromosomes and the X chromosome and < 1% in unassigned scaffolds (22,313 bp). The average depth of coverage mapping the 10× Chromium linked‐reads to the reference genomes was 24× for the Māui dolphin and 40× for Hector's dolphin (Table S1). The miniBUSCO score derived from the Cetartiodactyla database was 96.35% for the Hector's dolphin genome and 96.86% for the Māui dolphin, of which 0.99% and 1.28% were duplicated in the Hector's and Māui dolphin, respectively, and 0.68% and 0.52% were fragmented in the Hector's and Māui dolphin, respectively. The BUSCO scores of both the Hector's and Māui dolphin final assemblies are similar to the cetacean reference genomes utilised during assembly (Table S2).

**FIGURE 2:**
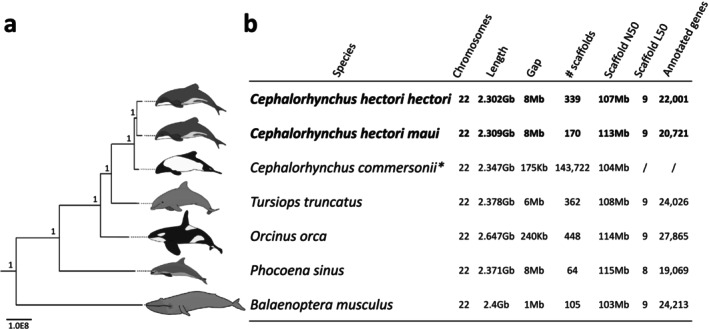
(a) SANS whole‐genome phylogenetic tree of the cetacean reference genomes used for scaffolding and the genomes assembled for the Hector's and the Māui dolphin, node labels with bootstrap values and scalebar indicating the k‐mer distance. (b) Hector's and Māui dolphin genome statistics showing consistency with VGP genomes (vaquita) and other high‐quality cetacean genomes. *The Commerson's dolphin assembly from the DNAZoo is chromosome‐length but not scaffolded into chromosomes.

The best quality genomes available for cetaceans, adhering to the VGP quality standards, are the vaquita, blue whale, bottlenose dolphin and killer whale. The reference genomes we obtained have comparable statistics, demonstrating the capability of our pipeline to generate high‐quality genomes despite less‐than‐optimal input samples (Figure 2b). Our phylogenetic tree also demonstrates the accurate placement of these two subspecies among cetacean species based on previous studies (Álvarez‐Carretero et al. 2022; McGowen et al. 2020) (Figure 2a).

**FIGURE 3:**
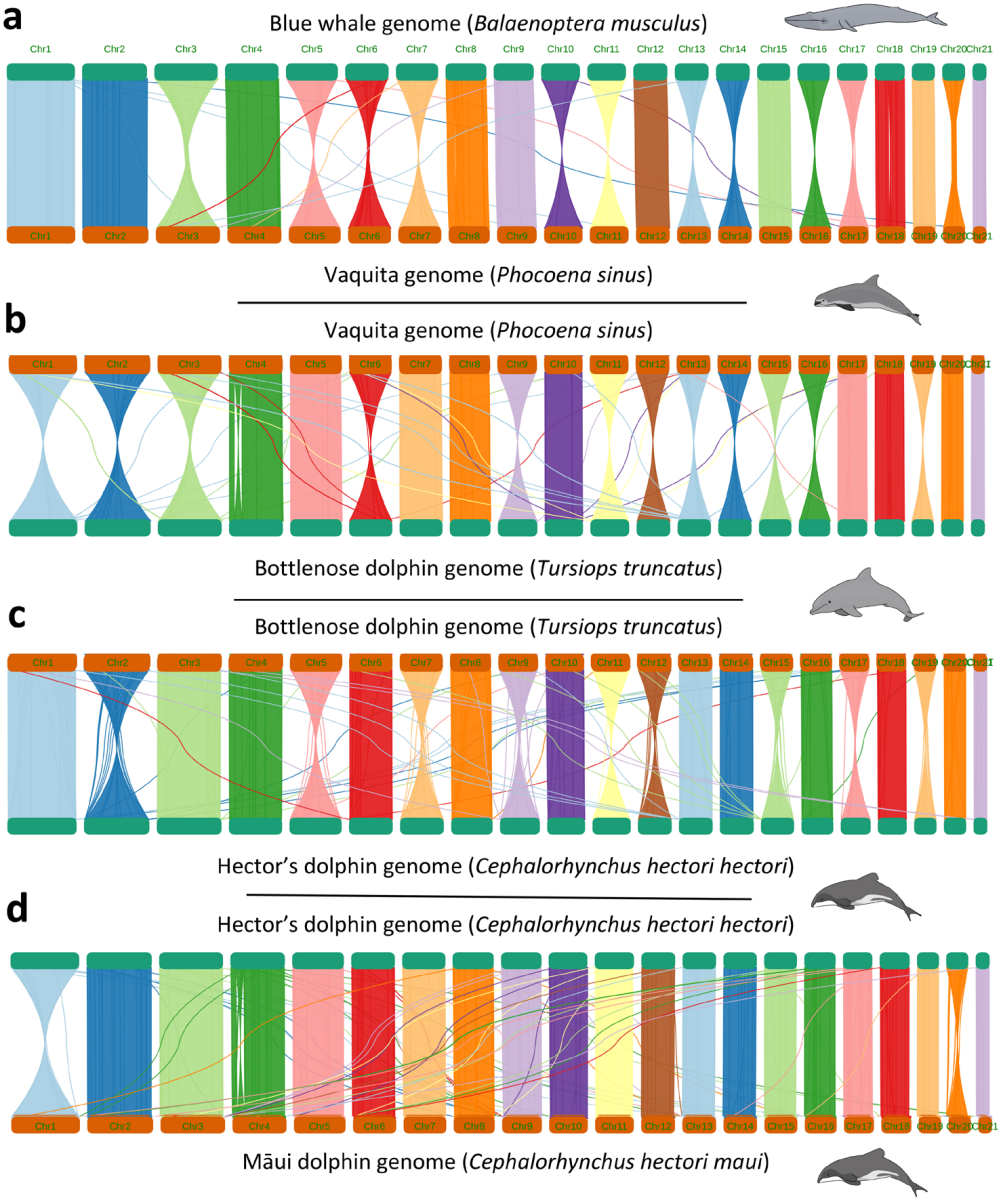
(a) Synteny between selected chromosome‐level cetacean genomes, ordered from species most evolutionary distant to Hector's/Māui dolphins (blue whale, see also Figure 2) to the subspecific comparison of the Hector's and Māui dolphin: (a) Synteny between the blue whale and the vaquita genome assemblies. (b) Synteny between the vaquita and the bottlenose dolphin genome assemblies. (c) Synteny between the bottlenose dolphin and the Hector's dolphin genome assemblies. (d) Synteny between the Hector's dolphin and the Māui dolphin genome assemblies.

The comparison of synteny plots revealed that some chromosomes were in the reverse orientation for some species, but overall demonstrated conserved synteny across all chromosomes, even for the distantly‐related blue whale and vaquita, supporting other recent findings (Morin et al. 2024). (Figure 3).

### 3.2 Genome Annotation

The percentage of repeats interspersed across the genome was 42.68% for the Hector's dolphin and 42.47% for the Māui dolphin, roughly consistent with the VGP vaquita genome (Table S3). Using the genomes soft‐masked for these repeats and combining two different annotation tools (BRAKER3 and GALBA) we identified 22,001 genes and 42,256 mRNAs in the Hector's dolphin genome and 20,721 genes and 39,569 mRNAs in the Māui dolphin genome. The longest gene and mRNA identified were similar for both dolphins, 498,158 and 498,185 bp, respectively. The mean gene length was higher for the Hector's dolphin at 32,104 bp compared to 24,569 bp for the Māui dolphin. The percentage of the genome covered by genes was higher for the Hector's dolphin at 22.5% compared to the Māui dolphin at 16% (Table S4). The number of annotated genes, total mRNAs, total introns, total exons and total CDS in comparison with the reference cetacean species was lower for both dolphins and lowest for the Māui dolphin (Table 1). The gene validation analysis classified 20%–30% of the predicted genes as good predictions (Table S5). The complete protein BUSCO was 81.1% for the Hector's dolphin genome annotations and 65.9% for the Māui dolphin (Table S6).

### 3.3 Historical Demography

**FIGURE 4:**
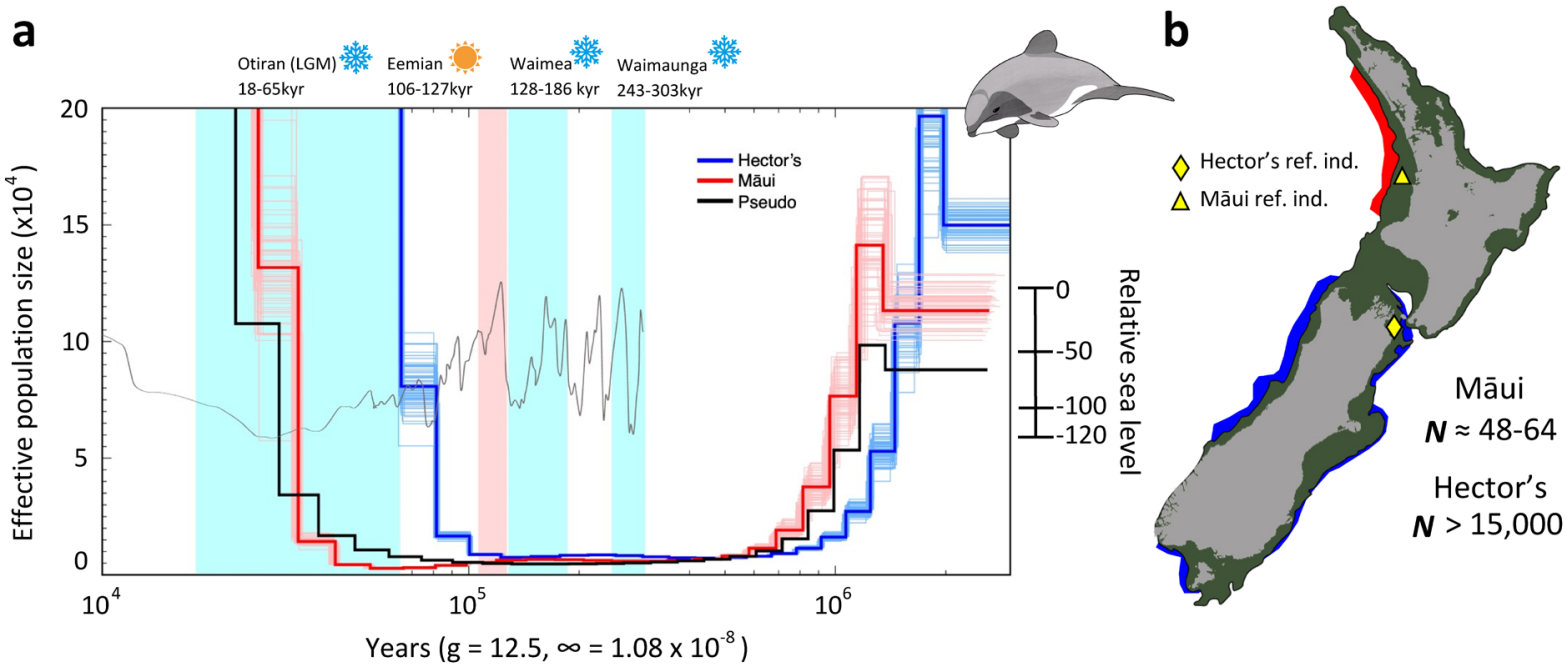
(a) Hector's (blue) and Māui (red) dolphin demographic reconstructions based on autosomal chromosomes with PSMC. Demographic estimates utilised a generation time of 12.5 years for both subspecies and an autosomal mutation rate of 1.08 × 10^−8^ substitutions per site/generation. *N* = 100 bootstrap replicates are shown by narrower lines. The pseudodiploid graph (black) was obtained using the same generation time and mutation rate. Glacial periods and the Eemian warm period are shown with light blue and light red, respectively, with names of periods given above the plot. The grey line reflects the relative to the present sea level increase and decrease in the past 500,000 years. (b) Reconstruction of New Zealand shoreline 20,000 years ago during the Last Glacial Maximum shown by dark green shading (modified from https://niwa.co.nz), current distribution of Hector's (blue) and Māui (red) dolphins, location of the individuals sampled for the reference genomes (yellow shapes), and current population estimates (Braulik et al. 2023; Slooten and Dawson 2021; Constantine et al. 2021) are also displayed.

The PSMC analysis demonstrated that Hector's and Māui dolphins have low long‐term effective population sizes (*N*_e_). Effective population size was stable for ~700,000 years from 800,000 to 100,000 years ago, at 20,000–30,000 effective individuals (Figure 4a). Following this period, there was an apparent increase in *N*_e_, however, PSMC plots can be affected by the emergence of population structure, in which case the y‐axis can be misinterpreted as a change in *N*_e_ instead of as the inverse of the coalescent rate (Cahill et al. 2016; Mather et al. 2020; Mazet et al. 2015, 2016; Morin et al. 2018). Therefore, the very rapid increase in *N*_e_ following the Eemian warm period likely reflects the emergence of population structure within Hector's dolphin populations, as gene flow was enabled by warm water corridors and habitat availability (Figure 4a).

We also generated a pseudodiploid genome from the Hector's and Māui dolphin genomes. The rate of coalescence within the pseudodiploid genome will decline forward in time, increasing to infinite *N*_e_ when there is a cessation of gene flow and complete isolation of the two populations. The Hector's–Māui dolphin pseudodiploid indicates that the divergence of these subspecies could have started during the start of the last glacial period (Otiran) 65 kya ago, when there was a gradual increase in N_e_, reflecting a potential reduction in gene flow between the subspecies (Figure 4a). The pseudodiploid plot further suggests that the two subspecies became completely isolated in the Last Glacial Maximum 20 kya ago, coincident with an emergence of population structure in the Māui dolphin population and large‐scale changes in ocean connectivity due to sea level lowering (Figure 4b).

### 3.4 Genome‐Wide Heterozygosity

**FIGURE 5:**
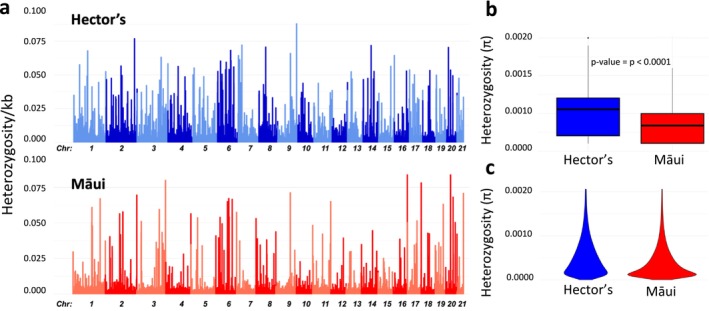
Distribution of heterozygosity across the Hector's and Māui dolphin genomes. (a) Per window heterozygosity in non‐overlapping 10 kbp windows with 2.5 kbp steps across the 21 autosomal chromosomes. (b) Comparison of mean genome‐wide heterozygosity of Hector's and Māui dolphin genomes. (c) Violin plot comparing the distribution of heterozygosity values across the 10kbp windows between Hector's and Māui dolphins genomes.

When examining heterozygosity across the 21 autosomal chromosomes, we observed a consistently higher level in almost all 10 kbp non‐overlapping windows for Hector's dolphin (mean genome‐wide heterozygosity = 0.0011, standard deviation = 0.0002) compared to the Māui dolphin (mean = 0.00070, standard deviation = 0.0002, statistically significant Mann–Whitney *U* test, *p* < 0.0001) (Figure 5). In addition to mean genome‐wide heterozygosity being approximately 40% higher in the Hector's dolphin than in the Māui dolphin, there was a more uniform distribution of heterozygosity across the Hector's dolphin genome. In contrast, the Māui dolphin showed low heterozygosity in multiple regions across all chromosomes, resulting in a distinctive sawtooth‐like pattern (Figure 5).

**FIGURE 6:**
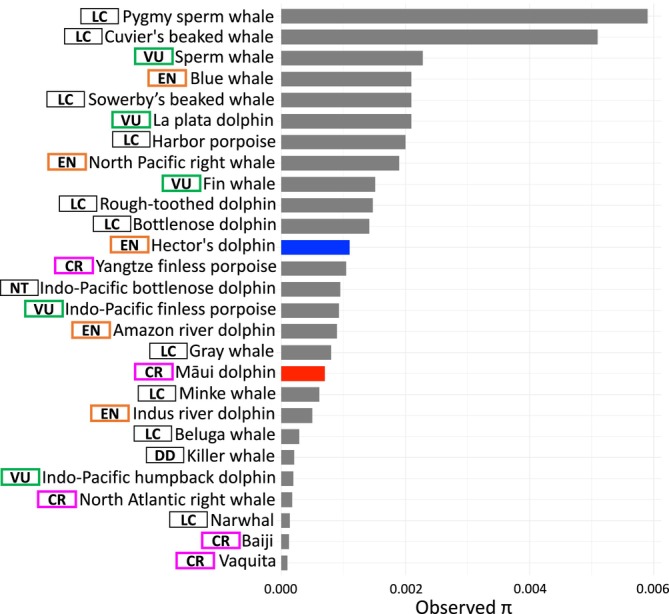
Genome‐wide heterozygosity values in cetacean species obtained from single genomes. Values obtained from this study, Huang et al. (2023) and Crossman et al. (2023). Hector's dolphin (blue bar), Māui dolphin (red bar), and other cetacean species (grey bars). IUCN conservation status is represented in the boxes, critically endangered (CR) in pink, endangered (EN) in orange, vulnerable (VU) in green, least concern (LC), non‐threatened (NT) and data deficient (DD) in black.

When compared to heterozygosity levels in other cetaceans, Hector's and Māui dolphins did not exhibit extreme values (Figure 6). However, the substantial difference in genome‐wide heterozygosity we observed between the Hector's and Māui dolphin genomes and the saw‐tooth‐like pattern across the genome suggests that Māui dolphins are at risk of loss of genetic diversity.

## 4 Discussion

In this study, we demonstrated the use of a reference‐guided scaffolding approach to achieve the assembly of high‐quality genomes, even when working with less‐than‐optimal input data.

In this case, our less‐than‐optimal input (tissues in ethanol stored between −20°C and 4°C for long periods of time) resulted in different DNA quality and a different Nanopore sequencing approach for each dolphin. Despite limits on DNA quality and quantity, utilising our synteny pipeline, we assembled two of the most complete cetacean genomes currently available (successfully mapping and scaffolding over 99% of the genome into chromosomes) (Whibley et al. 2021). Our ability to achieve these results was greatly facilitated by the existence of previously published high‐quality cetacean genomes, most notably the vaquita genome (Morin et al. 2021). The genomes of Hector's and Māui dolphins appear similar in quality to those of previously published ‘platinum standard’ VGP cetacean genomes, with some metrics even surpassing these previously published reference genomes (Morin et al. 2020). However, despite the success of our pipeline in the genome assembly, the lack of RNA data negatively impacted annotation for these genomes. An additional impact of DNA quality was observed, with the Hector's dolphin assembly (which had higher molecular weight input DNA) having better annotation than the Māui dolphin.

Obtaining these high‐quality genomes allowed us to perform subsequent analyses to determine the historical demography and genome‐wide heterozygosity of Hector's and Māui dolphins, demonstrating the utility of this reference‐based scaffolding approach for conservation‐relevant insights.

### 4.1 Utilising Closely Related Species to Improve Genome Assemblies

The success of our reference‐based scaffolding approach is driven by the remarkable karyotype conservation among cetaceans, which ranks among the most conserved of all mammalian orders (Árnason 1974; Árnason et al. 1984). This is evidenced by the synteny plots of selected chromosome‐level cetacean genomes, showing strong chromosomal conservation even between distantly related species within Cetacea, such as the blue whale (Mysticeti) and the vaquita (Odontoceti) (Árnason et al. 1984; Ferguson‐Smith and Trifonov 2007; Morin et al. 2024). Nearly all cetacean species, except for the sperm whale ( *Physeter macrocephalus* ), pygmy sperm whale ( *Kogia breviceps* ), and dwarf sperm whale ( *Kogia sima* ) (2*n* = 44), maintain a consistent karyotype of 2*n* = 46 (Árnason 1974). Furthermore, detailed analyses within the Delphinidae family consistently demonstrate a high degree of similarity in overall chromosome morphology (Brookwell et al. 2021), a pattern also evident in the synteny plot comparing the bottlenose dolphin and Hector's dolphin. This makes the reference‐based approach for chromosome scaffolding a robust and feasible strategy in Cetacea. However, the pipeline we demonstrate here is not restricted to only cetaceans, as it can be applied to any taxonomic group characterised by a similarly conserved and uniform karyotype across species, for example, feliforms (Adega et al. 2018; Perelman et al. 2005), pinnipeds (Beklemisheva et al. 2016), marsupials (Deakin et al. 2013; Rens and Ferguson‐Smith 2010), birds (Takagi and Sasaki 1974; Waters et al. 2021), and reptiles such as crocodilians and turtles (Deakin and Ezaz 2019; Waters et al. 2021). Our approach may prove particularly useful for taxa where multiple high‐quality, chromosome‐level reference genomes are available. For the Hector's and Māui dolphins, using multiple high‐quality, chromosome‐level reference genomes from closely related species outperformed solely using a non‐chromosome‐level, yet congeneric genome ( *Cephalorhynchus commersonii* ). The use of multiple genomes allowed for more accurate scaffolding and better overall assembly quality, potentially alleviating the influence of misassemblies within any one reference genome, so we would recommend this approach, if possible, in other taxa.

### 4.2 Long‐Term Low Effective Population Sizes in the Hector's and Māui Dolphin, Impacts on Genetic Diversity, and Implications for Inbreeding Susceptibility

Although our PSMC results suggested that both subspecies maintained a long‐term small *N*_e_ for ~700,000 years despite the multiple glacial periods in the Pleistocene, we observed changes in N_e_ associated with the Eemian warm period. We suggest that the apparent changes in N_e_ resulted from genetic interchange and gene flow between Hector's dolphin ( *Cephalorhynchus hectori hectori* ) subpopulations, as more habitat and warm‐water corridors became available due to increasing sea level, as seen in other marine megafauna (Bentley et al. 2023). Increased sea temperature can be associated with increased movement and altered distribution in highly mobile animals such as marine mammals (Kaschner et al. 2011; Schumann et al. 2013). In addition to the direct impacts of temperature on seascape connectivity, the Eemian warm period could have profoundly impacted prey availability (Brierley and Kingsford 2009), potentially resulting in increased home ranges and colonisation of new areas and/or the potential for gene flow with adjacent populations (Poloczanska et al. 2016).

Similar processes that led to genetic structure within Hector's dolphins seem to have driven the emergence of structure between the Hector's and Māui dolphins, which started in the interglacial period, with gene flow between the two subspecies ceasing in the LGM. During the LGM, sea level was around 120 m below today's and *Te Moana‐o‐Raukawa* (The Cook Strait) was completely closed, resulting in a physical barrier for interchange between East Coast Hector's dolphin populations in *Te Waipounamu* (South Island) and the Māui dolphin population of *Te Ika‐a‐Māui* (the North Island) (Hamner et al. 2014; Lewis et al. 1994; Proctor and Carter 1989). Subsequent apparent increases in Māui dolphin N_e_ could be linked to similar processes, with temperatures impacting population connectivity between the two subspecies and the characteristic philopatry of the species, resulting in the isolation of local populations (Pichler et al. 1998). Overall, we found that Hector's and Māui dolphin historical demographies are more similar to the vaquita than to any other delphinid (Huang et al. 2023; Morin et al. 2021; Westbury et al. 2023). The vaquita and the Hector's and Māui dolphins have similarly limited distributions. All are coastal cetaceans, endemic to relatively small areas of suitable habitat, which may have contributed to consistent long‐term low effective population sizes.

Such long‐term low effective population sizes, as observed in the vaquita, are not always a risk factor. While low genetic diversity is associated with increased extinction risk, its impact on conservation status can vary depending on ecological and demographic factors (Jeon et al. 2023; Huang et al. 2023; Morin et al. 2021; Robinson et al. 2016). In fact, when species have long‐term small effective population sizes for long periods of time, deleterious alleles can be purged, reducing the risk of inbreeding depression (Hedrick and Garcia‐Dorado 2016; Hedrick 1994; Orton et al. 2024). For example, genome‐wide heterozygosity of vaquita is the lowest reported for any whale, dolphin or porpoise, but the heterozygosity is relatively even across the whole genome, suggesting a limited impact of inbreeding depression, despite the extremely low population size (12 individuals) (Morin et al. 2021; Robinson et al. 2022).

In contrast, when populations with larger long‐term effective population sizes suffer a population bottleneck, deleterious alleles can become fixed, leading to inbreeding depression (Robinson et al. 2016, 2019, 2021, 2022). We observed a 40% lower genome‐wide heterozygosity and a saw‐tooth‐like uneven pattern of heterozygosity distribution in the Māui dolphin, in comparison with the Hector's dolphin. This uneven pattern, characterised by long ROH interspersed with regions of high heterozygosity, has previously been characterised as evidence of recent inbreeding depression in other species (Robinson et al. 2019, 2021). This pattern suggests that Māui dolphins are at risk of inbreeding which could have been caused by their isolation and reduction in genetic interchange with the Hector's dolphin during/following the LGM, compounded by anthropogenic impacts and the measured decrease in *N*_e_ in the last 15 years in this already very small and isolated population (Constantine et al. 2021). However, it is important to emphasise that our results are based on a single genome approach and should be followed up with population‐wide analysis using genomes of multiple individuals for additional evidence of the potential impacts of inbreeding depression.

## 5 Limitations

Our study demonstrates the successful use of synteny to generate high‐quality genomes from suboptimal samples; however, we acknowledge the limitations of using lower‐quality tissues for genome assembly. Our findings highlight the importance of prioritising tissue preservation strategies that maximise the likelihood of successfully extracting both DNA and RNA, particularly in conservation efforts where resources are limited (Williams 2010). Additionally, since our synteny‐based approach includes a *de novo* assembly step before the reference scaffolding process, we strongly recommend incorporating long‐read sequencing. Long reads are essential for resolving highly complex genomic regions and have been proven to significantly enhance the accuracy and continuity of *de novo* assemblies (Amarasinghe et al. 2020). When tissue quality, sequencing and computational resources are not constraints, we recommend following the Vertebrate Genomes Project (VGP) guidelines for assembling reference genomes (Rhie et al. 2021). While we recommend using multiple reference genomes to minimise scaffolding artefacts caused by errors or non‐syntenic rearrangements in any single reference, utilising a single high‐quality reference genome would still be valuable for providing synteny information to guide scaffolding. Although this approach carries a higher risk of introducing errors if the reference contains misassemblies or species‐specific rearrangements, it can still enhance genome contiguity and structure compared to relying solely on the draft assembly. Therefore, while our findings support the use of multiple reference genomes for optimal accuracy, a single well‐curated reference genome remains a viable option when necessary. The primary limitation of our genome assemblies was the absence of RNA data for Hector's and Māui dolphins, which affected the annotation process. High‐quality RNA data from these subspecies would enable more comprehensive genome annotation, improving our understanding of their genetic architecture.

## 6 Conclusions

The genome assembly pipeline presented here provides an opportunity to obtain high‐quality genome assemblies in other species of interest, even when samples, resources and DNA quality are not ideal. The reference genomes obtained for the critically endangered Māui dolphin and the endangered Hector's dolphin rival the quality of the most complete cetacean genomes published. Along with our assessment of heterozygosity and historical demography, these genomes will continue to contribute to a comprehensive understanding of the evolutionary history and genetic consequences of anthropogenic activities on these dolphins, while also providing genomic resources to facilitate ongoing conservation genomics research. Many long‐term tissue archives across various taxa have been subject to suboptimal storage conditions, often lacking the ideal temperature due to resource constraints (i.e., availability of −80°C freezers), or due to remote collection areas and long transport times. Consequently, the approach outlined in this study serves as a valuable resource for assembling reference genomes of taxa with long‐term collections exhibiting these characteristics.

## Author Contributions

A.A. conceptualised this study, with input from C.S.B., D.H., R.C., N.J.G., R.M.H., W.Ro. and D.H. A.A., N.J.G. and L.U. obtained funding. A.A. carried out consultation and engagement with *iwi* and *hapū*, with guidance from B.T.A. A.A., S.F. and J.Gi. conducted the lab work. S.A.‐C., L.U. and A.A. conducted analyses, with guidance from E.L.C., J.Gu., L.D. and W.Ra. S.A.‐C. wrote the manuscript, with extensive editorial input from A.A. A.A. translated the abstract into te reo Māori. All co‐authors provided comments and edited the manuscript.

## Conflicts of Interest

The authors declare no conflicts of interest.

## Benefit‐Sharing Statement

The data produced in this study will be useful for future research on the critically endangered Māui dolphin and the endangered Hector's dolphin and are currently being implemented in conservation workflows. Along with the published data, the publicly available genome assembly pipeline used in this paper provides a new approach to genome assemblies of different animal groups with conserved karyotypes via reference scaffolding. The Hector's and Māui dolphin reference genomes and genome annotation files are available via the Aotearoa Genomic Data Repository, which allows *rangatiratanga* (sovereignty) over this data to be maintained by *iwi* and *hapū*, and results have been communicated back to *iwi/hapū* in an accessible format (https://figshare.com/articles/presentation/Plain_speak_summary_of_the_Hector_s_and_M_ui_dolphin_genome_paper_Leveraging_synteny_to_generate_reference_genomes_for_conservation_Assembling_the_genomes_of_Hector_s_and_M_ui_dolphins_/27048361?file=49253878), including a visual guide (https://figshare.com/articles/presentation/Visual_guide_to_generation_of_Hector_s_and_M_ui_dolphin_reference_genomes_Leveraging_synteny_to_generate_reference_genomes_for_conservation_Assembling_the_genomes_of_Hector_s_and_M_ui_dolphins_/27048358?file=49253875). *Iwi/hapū* were also given the opportunity to contribute to the narrative as authors of the manuscript if they wished, and to receive a *koha* (a gift given in appreciation) in acknowledgement of their time. Finally, the abstract has also been translated into *te reo Māori* (the Māori language).

## Supporting information


Appendix S1.

## Data Availability

The data generated in this study (10× linked read FASTQ, ONT FASTQ, PCR‐free WGS FASTQ, genome assembly FASTA) and annotation tracks for both Hector's and Māui dolphin as specified in Table S7 can be accessed through the Aotearoa Genomic Data Repository: Māui reference genome—AGDR00067 https://doi.org/10.57748/r07b‐1j46, Hector's reference genome—AGDR00058—https://doi.org/10.57748/gzss‐j155, (Alvarez‐Costes et al. 2024). The Aotearoa Genomic Data Repository is designed to facilitate the sharing of *taonga* (treasured) non‐human genomic data originating in Aotearoa New Zealand while also ensuring the principles of Māori Data Sovereignty are respected (Te Aika et al. 2025). Therefore, this repository is emerging as a useful repository for a variety of recently published cetacean genomes originating from Aotearoa, New Zealand (Reeves et al. 2023; Foote et al. 2023; McGrath et al. 2024). Conditions and protocols for access are outlined at the DOIs above. All scripts for the analysis conducted in this study are available at https://github.com/sebasalco/GenomeSynteny_HectorsMauiRefGenomes
