# Supplementary material for: Leveraging Synteny to Generate Reference Genomes for Conservation: Assembling the Genomes of Hector's and Māui Dolphins
Source: Mol Ecol Resour. 2025 Apr 4;25(7):e14109. doi: 10.1111/1755-0998.14109 (PMC12415809; doi:10.1111/1755-0998.14109)

MOLECULAR ECOLOGY RESOURCES

Supplemental Information for:

Leveraging synteny to generate reference genomes for conservation: Assembling the genomes of Hector's and Māui dolphins.

Alvarez-Costes, S., Baker, C. S., Constantine, R., Carroll, E. L., Guhlin, J., Dutoit, L., Ferreira, S., Heimeier, D., Gemmell, N. J., Gillum, J., Hamner, R. M, Rayment, W., Roe, W., Te Aikā, B., Urban, L., and Alexander, A.

Table of Contents:

**Supplementary Table 1**	Page 1-2
**Supplementary Table 2**	Page 3
**Supplementary Table 3**	Page 3
**Supplementary Table 4**	Page 4
**Supplementary Table 5**	Page 4
**Supplementary Table 6**	Page 5
**Supplementary Table 7**	Page 6
**Supplementary Figure 1**	Page 7
**Supplementary Figure 2**	Page 7

Supplementary Table 1. Improvement in genome quality from single technology assemblies (10× and Nanopore) to merged assemblies (Quickmerge), before and after gap closing and scaffolding. C = contig, S = scaffold. The BUSCO scores reported are against the eukaryote database. Tables include final assembly statistics; Merqury k-mer based genome assembly evaluation statistics; and ONT mapping quality before and after reference-based scaffolding.

Assembly statistics of 10×, nanopore and merged assemblies before polishing

Global statistics	Hectors_10×	Hectors_Nanopore	Hectors_Merged	Maui_10×	Maui_Nanopore	Maui_Merged
BUSCO (%)	87.79	62.01	87.46	71.29	88.45	88.45
Length (bp)	2,364,075,673	1,951,136,765	2,365,778,208	2,285,900,071	2,318,907,597	2,328,510,593
Gap (bp)	19,880,100	0	19,283,170	82,723,560	0	29,307,230
Contigs (#)	14,474	47,268	12,968	53,914	9,579	5,257
C N50 (bp)	925,720	45,307	1,034,158	142,457	5,88,010	1,473,649
C L50 (#)	735	9076	666	10,663	1,214	435

Assembly statistics after polishing, scaffolding and gapclosing

Global statistics	Hectors_Scaff	Hectors_Scaff_Gagcl	Maui_Scaff	Maui_Scaff_Gapcl
BUSCO (%)	88.65	89.11	87.95	88.45
Length (bp)	2,324,792,008	2,324,762,454	2,314,615,039	2,314,609,758
Gap (bp)	14,280,230	7,989,915	22,792,180	8,733,930
Scaffolds (#)	2,128	2,128	614	614
S N50 (bp)	106,129,595	106,128,476	108,053,421	108,054,266
S L50 (#)	9	9	9	9

Final assembly statistics

Statistics	Hectors dolphin genome	Māui dolphin genome
Scaffolds (#)	339	170
bp in autosomes and X/Y	2,291,306,640	2,308,694,106
bp in unassigned scaffols	4,600,419	22,313
10x linked-reads coverage	40x	24x

Merqury QV assembly evaluation

Assembly	Hector's pre gap-closing assembly	Hector's final assembly	Māui pre gap-closing assembly	Māui final assembly
Quality Value (QV) score	**30.1338**	**30.6954**	**28.3512**	**28.5457**
Estimated error rate	0.000969653	0.000852039	0.00146178	0.00139774
Number of k-mers found in the genome assembly	1663158415	1665353842	1462850516	1471542461
Total k-mers from the sequencing reads	1719368598	1719368598	1517342052	1517342052
Genome completeness (%)	**96.7308**	**96.8585**	**96.4088**	**96.9816**

ONT mapping statistics before and after reference scaffolding

	Hector's pre-scaffolded assembly	Hector's final assembly	Māui pre-scaffolded assembly	Māui final assembly
Total reads	4,889,045	4,805,300	12,835,279	12,721,370
Mapped reads	4,636,191 (94.83%)	4,515,045 (93.96%)	12,095,505 (94.24%)	11,784,972 (92.64%)
Primary mapped reads	2,666,314 (91.34%)	2,628,913 (90.06%)	7,200,162 (90.68%)	7,003,538 (88.21%)
Secondary alignments	1,478,585	1,409,220	4,279,626	4,065,392
Supplementary alignments	491,292	476,912	615,717	716,042

Supplementary Table 2. BUSCO scores of all reference genomes and Hector's and Māui dolphin genomes. All statistics were obtained with the cetartiodactyla_odb10 (13335) database.

	Killer whale	Vaquita	Blue whale	Hector's	Māui
Complete BUSCOs	97.80%	98%	98.30%	97.34%	98.14%
Single-copy BUSCOs (S)	95.60%	96%	95.30%	96.35%	96.86%
Duplicated BUSCOs (D)	2.20%	2%	3.00%	0.99%	1.28%
Fragmented BUSCOs (F)	0.70%	0.50%	0.50%	0.68%	0.52%
Missing BUSCOs (M)	1.40%	1.50%	1.30%	1.98%	1.34%
N	13335	13335	13335	13335	13335

Supplementary Table 3. Repetitive content analysis in the Hector's and Māui dolphin genomes compared to the vaquita (Morin et al., 2021).

Repetitive content	Hector's	% of genome	Māui	% of genome	Vaquita	% of genome
Length	2,316,666,059		2,308,724,829		2,371,524,154	
SINEs	151,791,130	6.55	145,402,628	6.30	189,109,608	7.97
LINEs	606,339,498	26.17	603,305,047	26.13	653,546,597	27.56
LTR	111,172,343	4.80	111,829,528	4.84	134,757,334	5.68
Unclassified	82,477,127	3.56	82,967,970	3.59	1,047,864	0.04
Simple repeats	30,239,454	1.31	30,335,380	1.31	23,753,228	1
Low Complexity	6,721,490	0.29	6,669,935	0.29	4,527,734	0.19
Total	988,741,042	42.68	980,510,488	42.47	1,085,270,145	45.76%

Supplementary Table 4. Full genome annotation summary of Hector’s and Māui dolphins.

	Hector's	Māui
Total genes	22,001	20,721
Total mRNAs	42,256	39,569
Total introns	171,569	132,469
Total exons	149,568	111,750
Total CDS	42,315	39,600
Total gene length (bp)	518,938,908	374,216,370
Total mRNAs length (bp)	556,415,932	402,290,053
Total exon length (bp)	30,959,542	23,423,480
Longest gene (bp)	498,158	498,185
Longest mRNA (bp)	498,158	498,185
Longest exon (bp)	8,088	12,920
Mean gene length (bp)	32,104	24,569
Mean mRNA length (bp)	25,290	19,415
Mean exon length (bp)	180	177
% covered by genes	22.563	16.251
Mean mRNAs per gene	1.361	1.360
Mean exons per mRNA	7.798	6.393
Mean introns per mRNA	6.798	5.393

Supplementary Table 5. GeneValidator predictions scores of Hector’s and Māui dolphin genome annotations.

	Hector's	Māui
predictions	22,001	20,721
good_predictions	6,016	4,480
bad_predictions	15,985	16,241
predictions_w_insuff_blast_hits	5,148	5,287
first_quartile_of_scores	45	0
second_quartile_of_scores	64	45
third_quartile_of_scores	90	67

Supplementary Table 6. Protein BUSCO summary of Hector’s and Māui dolphin genome annotations.

Hector's: C:81.1%[S:58.4%,D:22.7%],F:3.1%,M:15.8%,n:255
Māui: C:65.9%[S:44.3%,D:21.6%],F:8.2%,M:25.9%,n:255

	Hector's	Māui
Complete BUSCOs	81.1% (207)	65.9% (168)
Complete and single-copy BUSCOs (S)	58.4% (149)	44.3% (113)
Complete and duplicated BUSCOs (D)	22.7% (58)	21.6% (55)
Fragmented BUSCOs (F)	3.1% (8)	8.2% (21)
Missing BUSCOs (M)	15.8% (40)	25.9% (66)
Total BUSCO groups searched	255	255

Supplementary Table 7. Raw data and genome files associated with the manuscript, available through The Aotearoa Genomic Data Repository.

Assembly	Size (bytes)	Name of file	Experimental strategy	Data type	Data format	Data category
Hectors_genome	2.48E+10	10X_Hectors_trimmed_clean_R1.fastq.gz	WGS	Unaligned Reads	FASTQ	raw read file
Hectors_genome	2.44E+10	10X_Hectors_trimmed_clean_R2.fastq.gz	WGS	Unaligned Reads	FASTQ	raw read file
Hectors_genome	16409	Hectors_mitogenome.fasta	Assembly	Mitogenome assembly	FASTA	processed file
Hectors_genome	1.00E+10	ONT_Hector2runsFiltered.fastq.gz	WGS	Unaligned Reads	FASTQ	raw read file
Hectors_genome	4478050506	WGS_Che11CB067_ME_R1_val_1.fq.gz	WGS	Unaligned Reads	FASTQ	raw read file
Hectors_genome	4705209442	WGS_Che11CB067_ME_R2_val_2.fq.gz	WGS	Unaligned Reads	FASTQ	raw read file
Hectors_genome	57766480	hectors_annotation.gtf	Annotations	Genome annotations	GTF	processed file
Hectors_genome	2169728983	hectors_aut_genome.fasta	Assembly	Genome assembly	FASTA	processed file
Hectors_genome	32920658	hectors_final_CDS.fasta	Annotations	Genome annotations	FASTA	processed file
Hectors_genome	11659344	hectors_final_proteins.fasta	Annotations	Genome annotations	FASTA	processed file
Hectors_genome	2316670371	hectors_genome.fasta	Assembly	Genome assembly	FASTA	processed file
Hectors_genome	1990656	Hectors_full_table_minibusco.tsv	BUSCO	BUSCO results	TSV	processed file
Maui_genome	2.01E+10	10X_Maui_trimmed_clean_R1.fastq.gz	WGS	Unaligned Reads	FASTQ	raw read file
Maui_genome	1.95E+10	10X_Maui_trimmed_clean_R2.fastq.gz	WGS	Unaligned Reads	FASTQ	raw read file
Maui_genome	16406	Maui_mitogenome.fasta	Assembly	Mitogenome assembly	FASTA	processed file
Maui_genome	1.12E+10	ONT_Maui6runsFiltered.fastq.gz	WGS	Unaligned Reads	FASTQ	raw read file
Maui_genome	1961127118	WGS_H267_ME_R1_val_1.fq.gz	WGS	Unaligned Reads	FASTQ	raw read file
Maui_genome	2074514683	WGS_H267_ME_R2_val_2.fq.gz	WGS	Unaligned Reads	FASTQ	raw read file
Maui_genome	56068661	maui_annotation.gtf	Annotations	Genome annotations	GTF	processed file
Maui_genome	2184003266	maui_aut_genome.fasta	Assembly	Genome assembly	FASTA	processed file
Maui_genome	25093330	maui_final_CDS.fasta	Annotations	Genome annotations	FASTA	processed file
Maui_genome	9007959	maui_final_proteins.fasta	Annotations	Genome annotations	FASTA	processed file
Maui_genome	2308726221	maui_genome.fasta	Assembly	Genome assembly	FASTA	processed file
Maui_genome	2097152	Maui_full_table_minibusco.tsv	BUSCO	BUSCO results	TSV	processed file

Supplementary Figure 1. Contamination reports of Hector’s and Māui dolphin merged assemblies. These contamination reports are *prior* to contaminant filtering using FCS-GX. a) Contamination report for Hector’s dolphin *de novo* merged assembly. b) Contamination report for Māui dolphin *de novo* merged assembly.

a

Pre-polishing Hector’s dolphin assembly contamination report

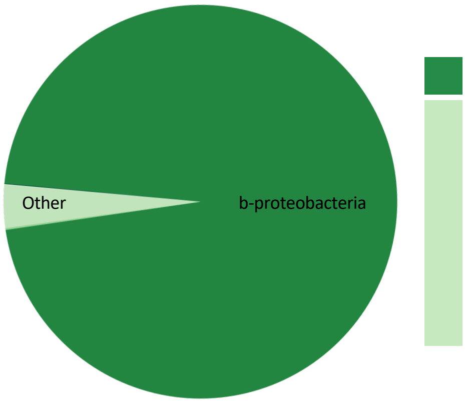

Taxonomic group	Length (bp)
prok:b-proteobacteria	6744626
anml:insects	247426
anml:nematodes	7884
virs:prokaryotic viruses	5385
anml:fishes	2621
anml:primates	2266
prok:a-proteobacteria	1875
Total (bp)	7012083

b

Pre-polishing Māui dolphin assembly contamination report

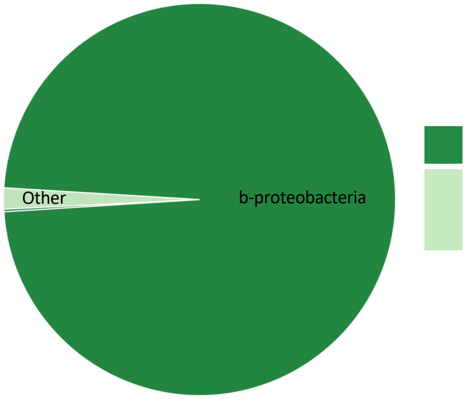

Taxonomic group	Length (bp)
prok:b-proteobacteria	2391446
prok:CFB group bacteria	42522
virs:prokaryotic viruses	5000
Total (bp)	2438968

Supplementary Figure 2. SANS whole genome phylogenetic analysis of Delphinidae species. Scale bar represent k-mer distances. Porpoises as outgroup. Database origin of each genome indicated by colour.

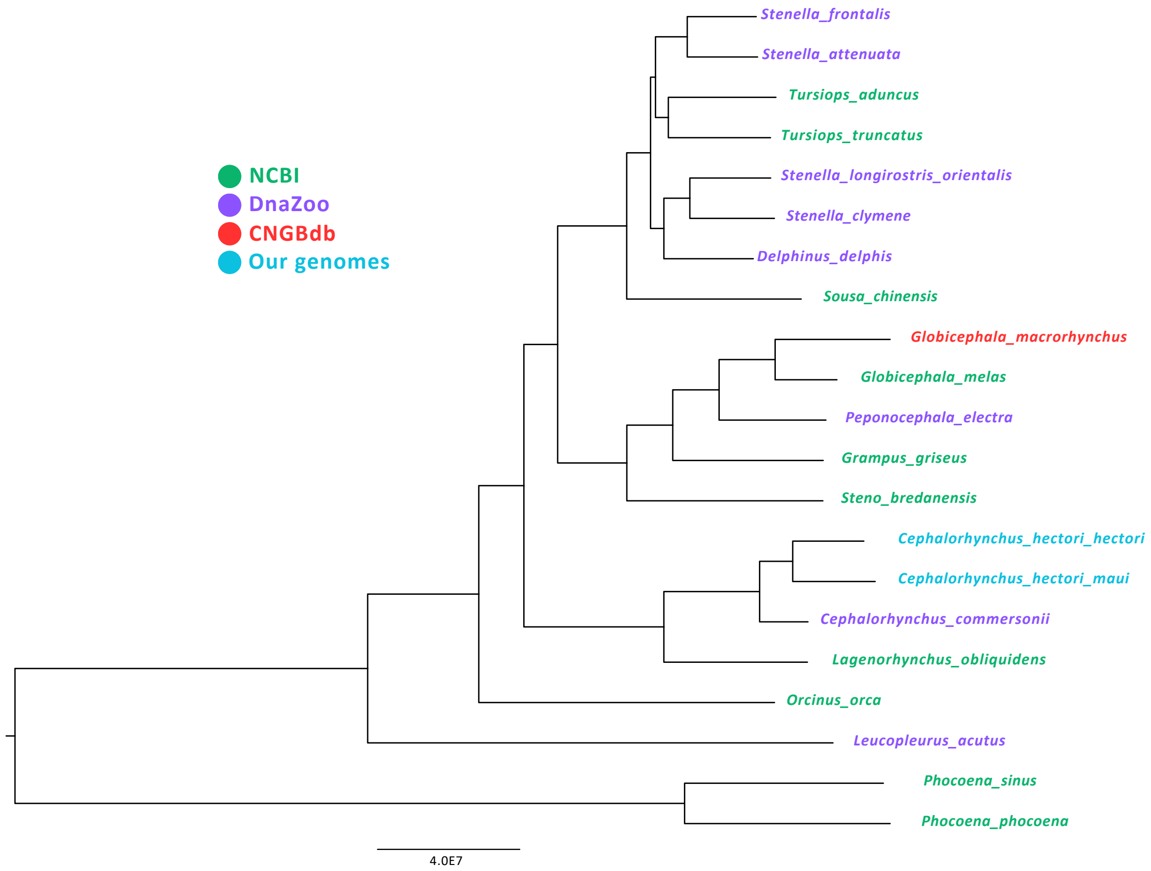

Supplement: Supplementary file 1 — Appendix S1. [file MEN-25-e14109-s001.pdf]
